# Evolutionary expression differences of creatine synthesis-related genes: Implications for skeletal muscle metabolism in fish

**DOI:** 10.1038/s41598-019-41907-6

**Published:** 2019-04-01

**Authors:** Andreas Borchel, Marieke Verleih, Carsten Kühn, Alexander Rebl, Tom Goldammer

**Affiliations:** 1 Fish Genetics Unit, Institute of Genome Biology, Leibniz Institute for Farm Animal Biology (FBN), Wilhelm-Stahl-Allee 2, 18196 Dummerstorf, Germany; 2 SLRC-Sea Lice Research Centre, Department of Biology, University of Bergen, Mailbox 7803, 5020 Bergen, Norway; 3 Institute of Fisheries, State Research Centre for Agriculture and Fisheries Mecklenburg-Western Pomerania (LFA MV), Fischerweg 408, 18069 Rostock, Germany

## Abstract

The creatine/phosphocreatine system is the principal energy buffer in mammals, but is scarcely documented in fish. We measured the gene expression of major enzymes of this system, glycine amidinotransferase (GATM), guanidinoacetate N-methyltransferase (GAMT) and muscle-type creatine kinase (CKM) in kidney, liver, and muscle tissues of fish and mammals. *CKM* was expressed strongly in the muscles of all examined species. In contrast, *GATM* and *GAMT* were strongly expressed in the muscle tissue of fish, but not of mammals. This indicates that creatine synthesis and usage are spatially separated in mammals, but not in fish, which is supported by RNA-Seq data of 25 species. Differences in amino acid metabolism along with methionine adenosyltransferase gene expression in muscle from fishes but not mammals further support a central metabolic role of muscle in fish, and hence different organization of the creatine/phosphocreatine biosynthesis system in higher and lower vertebrates.

## Introduction

**Figure 1:**
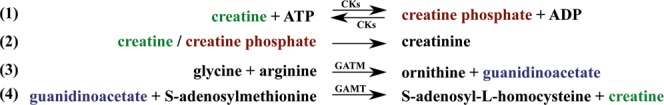
Reactions involved in the creatine system. The chemical equations represent (1) the Lohmann-reaction, (2) the formation of creatinine, (3) the first step of creatine synthesis and (4) the second step of creatine synthesis. Catalyzing enzymes are indicated at the arrows. Only the main reagents involved in the reactions are given.

Chemically-bound energy is the fuel of life. Adenosine triphosphate (ATP) is the primary supplier of energy. When ATP levels decrease, creatine phosphate regenerates the ATP stores by transferring a high-energy phosphate to adenosine diphosphate (ADP). The products of this reversible reaction are ATP and creatine. This so-called Lohmann-reaction (Fig. 1, equation 1) is catalyzed by creatine kinases (CKs) even before glycolysis and respiration start delivering energy^1^.

Essentially, creatine and its phosphorylated form represent an energy buffer that stabilizes and maintains cellular energy balance. Around 1.6% of the daily creatine/creatine phosphate pool spontaneously converts to creatinine (Fig. 1, equation 2), which cannot store energy anymore and is excreted^2^. The refill of creatine requires either nutritional uptake or endogenous synthesis. A typical US-American diet supplies around 50% of human creatine demands^3^, while vegans do not consume dietary creatine^4^. The demand for additional creatine has to be fulfilled by endogenous production. Creatine is synthesized by a two-step mechanism^5^ that involves the two enzymes glycine amidinotransferase (GATM, also known as AGAT) and guanidinoacetate N-methyltransferase (GAMT). In the first step (Fig. 1, equation 3), glycine and arginine form guanidinoacetate. In the second step (Fig. 1, equation 4), this compound and S-adenosyl-methionine (SAM) finally yield creatine.

In mammals, the individual steps of creatine synthesis and creatine usage are spatially separated ^reviewed in^^5^. While GATM is mainly active in mammalian kidneys, GAMT is most abundant in the liver. The creatine transporter CT1 imports creatine into tissues with creatine demand and is encoded by the *SLC6A8* gene. Creatine kinases are mainly found in tissues with high energy demands such as muscle and brain tissues. Deviating from this, it has been demonstrated that all enzymes required for the synthesis and usage of creatine are expressed in the brains of rats^6,7^. In accordance with the findings in rat brains, we recently suggested that the synthesis and usage of creatine are obviously not separated in rainbow trout also. Contrary to the expectations for *GATM* and *GAMT* expression in the kidneys and liver of trout, the skeletal muscle as profiteer of creatine comprises higher transcript amounts of these genes^8^. The present study examines whether this finding is unique for rainbow trout or if it is valid for other economically important fish species as well. We compare expression profiles of factors contributing to the creatine systems in animals from various clades of the tree of life such as mice (*Mus musculus*), cattle (*Bos taurus*) and pigs (*Sus scrofa*) as mammalian representatives and maraena whitefish (*Coregonus maraena*), pikeperch (*Sander lucioperca*), European perch (*Perca fluviatilis*) and Atlantic herring (*Clupea harengus*) as teleostean representatives. Additionally, we analyze gene-expression patterns from several other species based on publicly available RNA-Seq data to compare creatine systems between the vertebrate taxa *pisces*, *amphibia*, *reptilia*, *aves*, and *mammalia*.

## Results

### Identifying creatine-relevant genes in various species

We isolated >300-bp fragments of genes encoding GAMT, GATM, and CKM from four teleost species based on a PCR approach with degenerated primer oligonucleotides. The *GAMT* sequence from whitefish was complemented with contigs that were generated by transcriptome sequencing of maraena whitefish and was verified via PCR and DNA sequencing. Remarkably, GAMT is encoded by duplicated genes in whitefish, termed in the following as *GAMTa* and *GAMTb* (Supplementary Fig. S1). The coding domain of both variants is highly homologous with a sequence identity of 95%.

**Figure 2:**
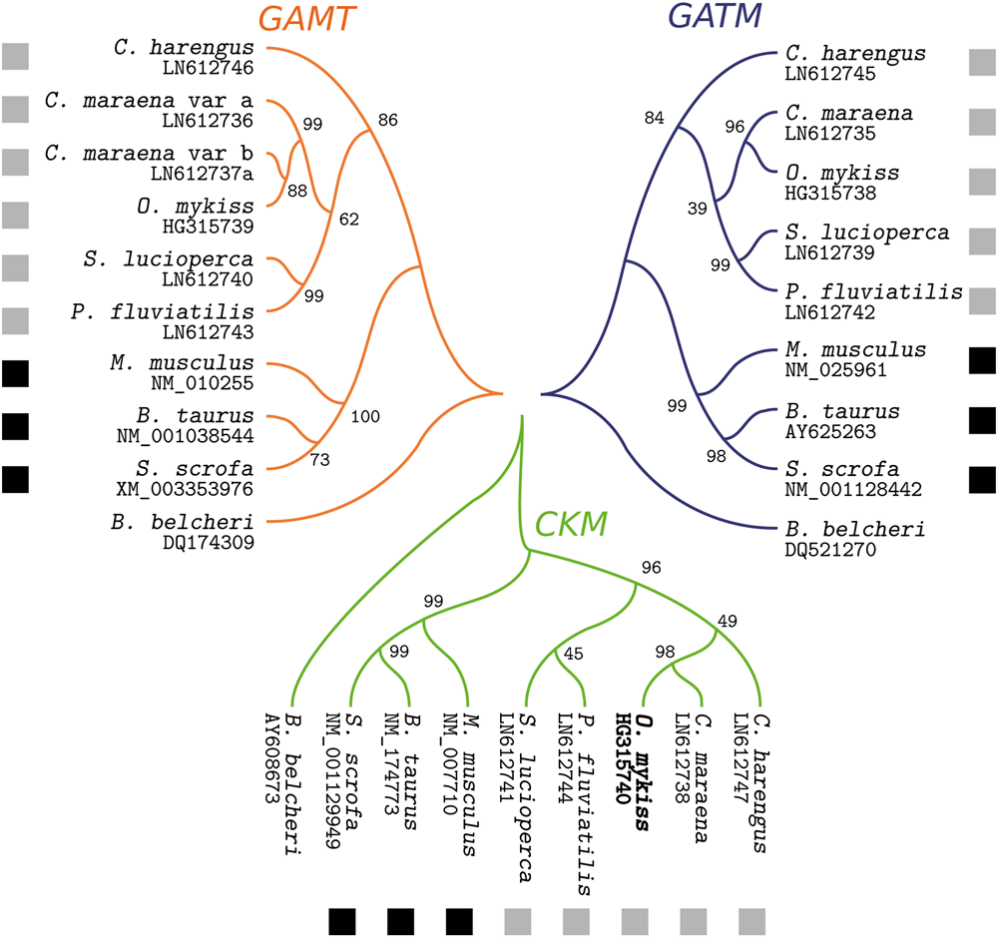
Phylogenies of partial nucleotide sequences of *GATM*, *GAMT* and *CKM*. *GATM*, *GAMT*, and *CKM* sequences from the amphioxus *Branchiostoma belcheri* were used as the outgroup. Bootstrap values are given at the branches. Gray squares mark piscine sequences, black squares mark mammalian ones.

We constructed phylogenetic trees based on the resultant partial cDNA sequences (Fig. 2). All three trees show separate clustering of the mammalian orthologs in one group and its piscine counterparts in another group, which agrees with the phylogenetic tree of life.

### Gene expression of *GATM*

**Figure 3:**
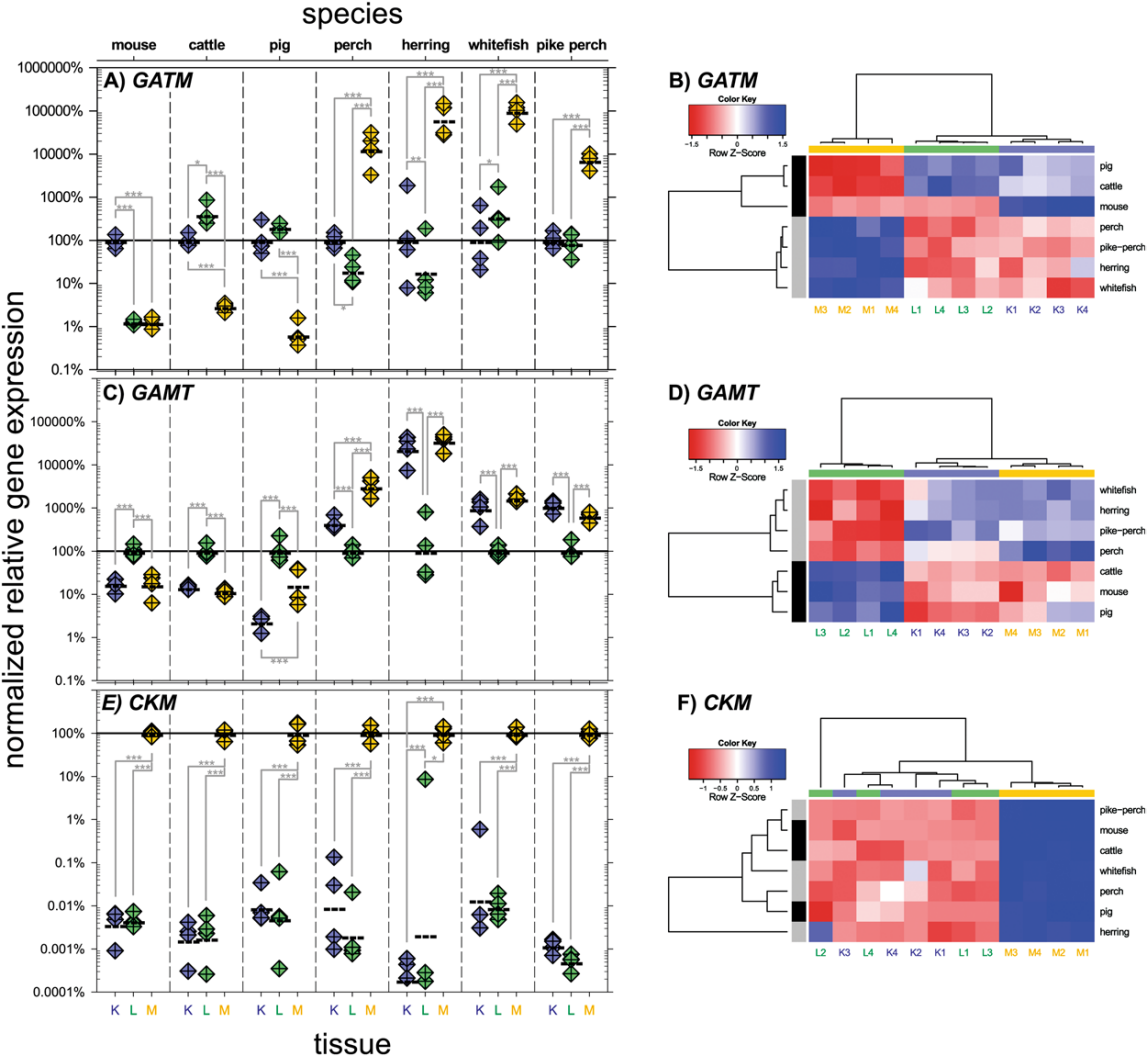
Expression levels of the genes *GATM*, *GAMT*, and *CKM* in kidney, liver, and muscle tissues of various species. (**A**,**C**,**E**) On the left-hand side, normalized expression levels are shown. The dotted lines indicate the geometric mean. *GATM* expression values were normalized separately for each species to its averaged expression in the kidneys; *GAMT* expression values were normalized to average hepatic expression and *CKM* expression values were normalized to average muscular expression. The maraena whitefish *GAMT* expression level is based on *GAMTa*. **(B**,**D**,**F)** On the right-hand side, heatmaps and clusters based on hierarchical clustering are shown. Blue and red fields indicate a high and low standardized (z-transformed) expression, respectively. Asterisks mark significant differences between groups (*p ≤ 0.05, **p ≤ 0.01, ***p ≤ 0.001). Species: Mouse (*Mus musculus*), cattle (*Bos taurus*), pig (*Sus scrofa*), European perch (*Perca fluvialtilis*), Atlantic herring (*Clupea harengus*), maraena whitefish (*Coregonus maraena*), pikeperch (*Sander lucioperca*).

Reports define the kidneys, liver, and muscles as the “classical” organs of creatine synthesis or usage in mammals. We first determined the copy number of *GATM* in these organs from three mammalian and four teleost species (Fig. 3A). All mammalian species showed a low *GATM* mRNA concentration in the skeletal muscle, whereas the kidneys (mice, cattle, pigs) and the liver (cattle, pig) displayed high *GATM* transcript levels. Inverse to this, the muscle tissue of all examined fish species exhibited an approximately 100-fold elevated *GATM* copy number compared to low *GATM* levels in the kidneys and liver. The corresponding cluster analysis (Fig. 3B) confirmed that the *GATM* expression profiles of all fishes form one cluster that is separated from those of mammals. In this respect, it is remarkable that within the group of mammals there is a large difference between cattle and pigs on one hand and mice on the other hand regarding hepatic *GATM* expression.

### Gene expression of *GAMT*

The expression patterns of *GAMT* (Fig. 3C) were similar in all examined mammals with high *GAMT* mRNA abundances in the liver and significantly lower mRNA levels in the kidneys and muscles. As observed for the *GATM* profiles, *GAMT*-transcript levels were low in the livers of all examined fishes, while the transcript levels in the kidneys and muscles were higher. In perch, the *GAMT* mRNA concentration in muscles exceeded the concentration in the kidneys, while it was the same in these organs in the other fishes. In whitefish, both *GAMT* isoforms showed similar expression patterns (Fig S1B) with a somewhat lower *GAMTb* level in the liver compared to the level of its paralog. The cluster analysis of *GAMT* profiles (Fig. 3D) again clearly separated fishes and mammals.

### Gene expression of *CKM*

The transcript level of *CKM* (Fig. 3E) was similar in all examined species. While copy numbers were low in the kidneys and the liver, the skeletal muscle displayed a strong *CKM* expression. The copy number differences between the tissues reached up to four orders of magnitude. The concordant patterns of *CKM* expression are reflected in the cluster analysis (Fig. 3F), which separated neither mammalian and teleostean species nor kidney and liver samples.

### RNA-Seq based expression levels

**Figure 4:**
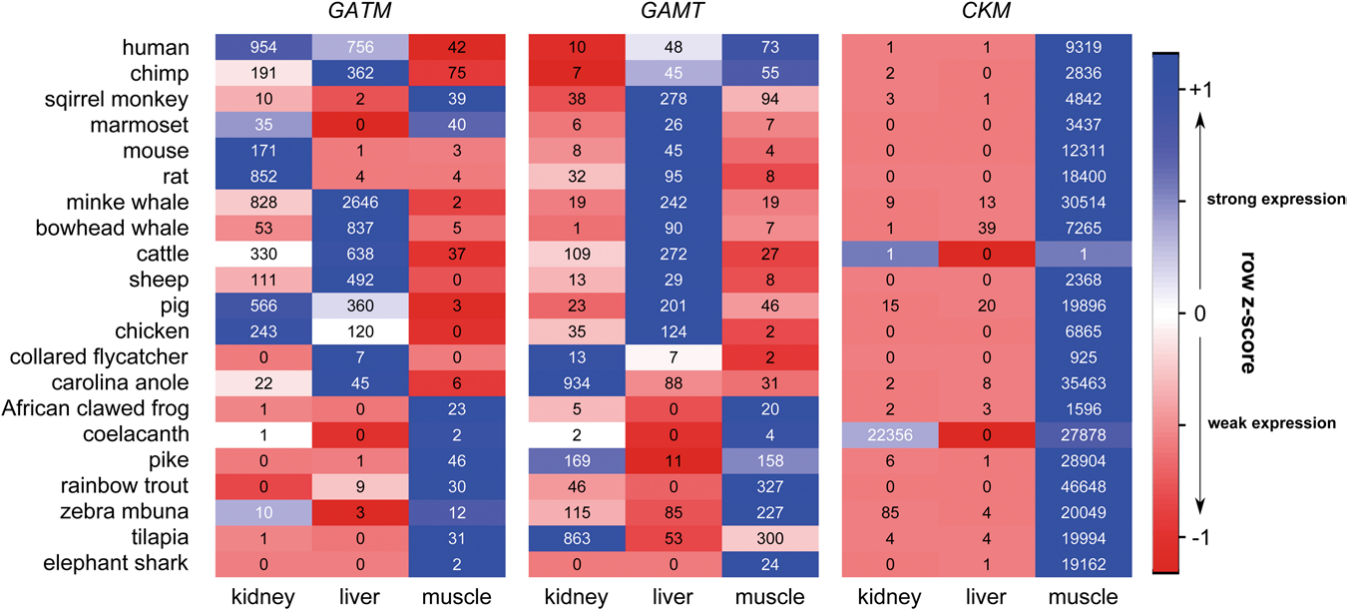
RNA-Seq based expression patterns of the genes *GATM*, *GAMT*, and *CKM* in kidney, liver, and muscle tissues of various species. Numbers are mapped reads per million total reads. Background colors encode the standardized expression values as row z-scores (even, smaller or higher than the mean expression value between tissue specific reads) for each gene and each species separately.

In addition to the qRT-PCR we analyzed publicly available RNA-Seq datasets in regard to the expression of creatine-related genes in kidneys, livers and muscles (Fig. 4). All of the examined fishes showed the same strong *GATM* expression pattern in the muscles and weak or no expression in the kidneys and liver. The same was true for the African clawed frog as well as the squirrel monkey and the marmoset. The other examined species showed only negligible *GATM* expression in the muscle tissue, but strong expression levels in the kidneys (pigs, chickens, rodents and human) and the liver (flycatchers, whales, cattle, sheep and chimps).

In regard to *GAMT* expression, all fish showed low or no hepatic expression but predominant muscular and renal expression. This also applies to the examined frog species. In contrast, the kidneys (anole, flycatcher) and liver showed high *GAMT* expression in other animals. The examined primates showed a high *GAMT* expression level in liver and muscle tissues.

### *CKM* expression was similar in all examined species, with the strongest expression in the muscles

Further, we analyzed the expression levels of *SLC6A8* in various vertebrates (Supplementary Table S1), but the profiles obtained differed not only between mammals and fish but also within the fish clade. In rats, humans and sheep, the highest expression was always found in the kidneys, followed by muscles and the liver, but this order differed between fish species. In zebra mbuna the highest expression was found in the kidneys, in pike in the muscles and in rainbow trout one *SLC6A8* gene copy was expressed mainly in the kidneys and the other in muscle tissues.

## Discussion

It has been well documented that mammalian muscles hardly produce creatine, but vitally depend on creatine/phosphocreatine^5,9^. In line with those reports, we detected a comparatively low muscular expression of the genes that encode the creatine-producing enzymes GATM and GAMT in three mammalian species (mice, cattle and pigs). Nevertheless, this expression data gives positive evidence for residual *GATM* and *GAMT* expression in mammalian muscle tissues, which confirms the findings of previous studies that describe limited creatine production in mammalian muscles^10–12^.

Only a few studies have examined the creatine system in teleost species so far. Wang and colleagues concluded that the expression patterns of *GATM* and *GAMT* in zebrafish and mammals are similar^13^. However, their investigations did not include muscle samples. A recent RNA-Seq dataset (Bioproject PRJNA255848) confirmed the expression of *GATM* (26 mapped reads/million reads in muscles; 0.5 in kidneys and 1 in the liver) and *GAMT* (182/28/162) in the muscles of zebrafish. We found high expression levels of genes encoding both enzymes necessary for creatine synthesis in the muscle samples of four fish species (maraena whitefish, pikeperch, European perch, and Atlantic herring) compared to kidney and liver, contrasting our findings in mammalian muscles. Two different interpretations appear possible. One explanation could be that the expression levels in kidney and liver of fish are reduced and that the muscular expression is in itself low, so that overall in none of these organs creatine synthesis takes place. Alternatively, the muscular expression of GATM and GAMT could be strong enough to deliver the creatine needed by the muscles. We think that the second alternative is more likely. On an absolute level, the copy number of *GATM* for example in pike muscle is in the same magnitude as in kidney and liver of cattle (Supplementary Fig. S2). For *GAMT* the piscine muscular mRNA copy numbers were higher than in RNA samples from mammalian kidneys. Additionally, it must also be kept in mind that muscles compose a much larger part of the body then kidneys or livers. If the creatine synthesis is decentralized, taking place directly at the site of usage, lower expression levels of the enzymes are sufficient then in the centralized case, where kidney and liver have to sustain all muscles of the body. Thereby the piscine muscle appears to be capable of synthesizing creatine autonomously and hence independent from creatine import, although this remains to be shown on a protein and metabolic level.

Especially *GATM*, the rate-limiting enzyme of creatine production^14^ is expressed more than 100-times stronger in the muscles than in the kidneys of bony fish. The high expression of *GATM* in muscles is especially surprising because high creatine levels (obtained by a creatine-rich diet) have been reported to decrease the transcription of *GATM* in rats^15,16^. The molecular background of this negative product feedback remains unknown. Piscine and mammalian muscles contain comparable amounts of creatine, as indicated by similar creatine content in meat products from fish and mammals^17^. If creatine-dependent regulation of *GATM* expression occurs in fish, muscular *GATM* expression should consequently be very low or absent. However, the rat pancreas is also capable of synthesizing creatine and expressing GATM as well as GAMT. Here, creatine supplementation that lead to an increased pancreatic creatine concentration did not have an effect on *GATM* transcript levels^18^, suggesting a different feedback regulation of GATM in rat kidneys and pancreas. It remains unclear why some but not all fish species had a high muscular expression of the creatine transporter SLC6A8 although they expressed it together with the genes necessary for creatine synthesis. Although it is not known yet how much creatine fish take up with their feed, it is conceivable that fish use muscular creatine transporters to transfer exogenous creatine from the blood stream into muscle cells to support endogenous creatine synthesis.

**Figure 5:**
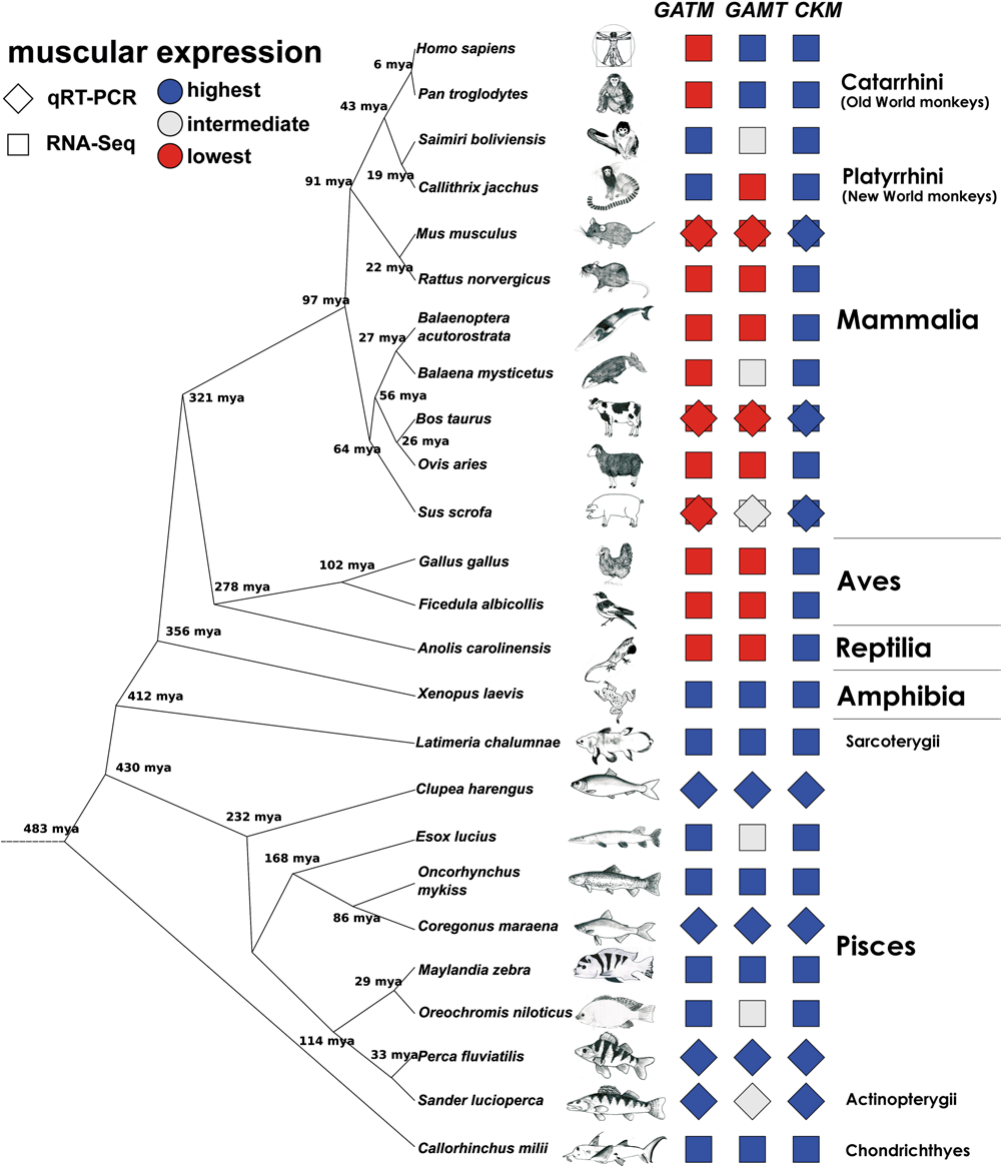
Animal species used in this study, their evolutionary relationship and muscular expression pattern of creatine-related genes. Data for the separation of species was taken from the TimeTree of Life project (http://www.timetree.org/). Squares show the strength of the expression of each gene. Blue squares indicate highest expression in the muscle in comparison to the liver and kidneys, white squares an intermediate expression between the other tissues and red ones the lowest expression. Rotation of the square indicates if data was gained from RNA-Seq or qRT-PCR. Animal names and taxonomy are based on the NCBI Taxonomy browser (www.ncbi.nlm.nih.gov/Taxonomy) and NCBI LinkOut pages, such as the Animal Diversity Web of the Museum of Zoology at the University of Michigan (www.animaldiversity.org). Human and animal art illustrations were kindly contributed by Luisa Falkenthal.

Muscular creatine synthesis seems to be a conserved feature among fishes. Figure 5 illustrates that the evolutionary distance between herring and pikeperch is more than twice as wide as the distance between mice and cattle, yet they do not differ in *GATM* expression. However, validation of the hypothesis that fishes synthesize a high-level and mammals’ a low-level of muscular creatine requires experimental proof.

In contrast to muscular creatine synthesis muscular creatine usage seems evenly distributed among all of the examined animal species. Muscles have a very high energy demand and accordingly up to 94% of the mammalian creatine content is found in muscles^5^. This is likely transferable to fishes, which need white muscles for highly energy-demanding burst-swimming^19^.

The evolutionary separation of animals synthesizing muscular creatine and animals synthesizing it elsewhere may coincide with the emergence of the mammalian species. Aligning the RNA-Seq data with the tree of life^20^ (Fig. 5) allows for a more precise estimate of the evolutionary shift of the tissue distribution of creatine-metabolizing enzymes. All fish shared the same pattern and also the frog *Xenopus laevis* showed high *GATM* and *GAMT* transcript levels in muscle tissues. In contrast, anoles showed only very low muscular expression of *GATM* and *GAMT*. We assume that the shift from local creatine synthesis in muscles to a more complex creatine system involving transport steps occurred in early amniotes, so this complex pattern can be found in reptiles, birds and mammals. However, within the group of primates a further shift seems to have taken place as two examined New-World monkeys show a high muscular *GATM* expression and chimpanzees and humans show a high muscular *GAMT* expression. We assume that creatine synthesis and usage in the same organ is the primordial mode of creatine-system organization. This is supported by the finding that the elephant shark *Callorhinchus milii*, a cartilaginous fish, also expresses genes required for creatine synthesis in its muscles. Overall tissue specificity of *GATM* and *GAMT* seems to have changed several times during evolution. Renal *GATM* expression seems to be very important only in rodents, while in ungulates and whales hepatic *GATM* expression seems to dominate. This raises the question of whether mice and rats are suitable model organisms for human creatine-deficiency syndromes which are caused by a lack of GATM, GAMT or the creatine transporter^21^.

Possible reasons for the different patterns of creatine synthesis in mammals and fish may reside in species-specific traits of amino-acid metabolism. The amino acids arginine and glycine are obligatory for the first step of creatine synthesis. Glycine is considered a conditionally essential amino acid for mammals^22^, whereas it is non-essential for fish^23^. Arginine is only semi-essential for mammals^24^, whereas it is essential for fish^23,24^. In other words, fish are dependent on the nutritional uptake of arginine^25^, which is then distributed via the blood stream throughout the body, probably to be directly utilized for creatine synthesis in muscles. In mammals where arginine is partly synthesized especially in kidney^26^ it may be beneficial to concurrently synthesize guanidinoacetate at this site as the necessary compounds are already available, thus reducing effort for arginine transport. Another component that is essential for the formation of creatine is the universal methyl-group donor S-adenosylmethionine (SAM), a co-substrate of GAMT. In humans, 40% of the methyl transfers from SAM are performed by GAMT in creatine synthesis^27^. SAM itself is produced by S-adenosylmethionine synthetase isozymes, encoded by the *MAT1A* (Methionine Adenosyltransferase 1A) and *MAT2A* genes. In mammals, most SAM is produced in the liver^28^. The expression atlas^29^ confirmed a dominant expression of *MAT1A* in mammalian livers, while no expression was observed in muscles. When searching for homologues in the PhyloFish database^30^ we found that fish from different taxonomic groups had at least two gene copies. One of which was expressed strongly in the liver, the other strongly in the muscle, as observed for brown trout (muscle-expressed: NST_LOC100697074.1.1; liver-expressed: NST_LOC101076112.2.2), European perch (MPF_LOC101487602.1.1; MPF_LOC101160342.1.1), Northern pike (HEL_LOC100697074.2.2; HEL_LOC101076112.1.1) and European eel (DAA_METK2.2.3; DAA_MAT1A.2.2). This suggests that fish, in contrast to mammals, might produce sufficient SAM in their muscles as a precondition for creatine synthesis. Apparently, all necessary enzymes involved in this process are expressed in piscine muscles.

In sum, the strong expression of genes coding for enzymes of the creatine-synthesis pathway in piscine skeletal muscles contrasts the negligible expression of the respective orthologs in mammalian skeletal muscles. This suggests that fishes are capable of producing significant amounts of creatine in their muscles, which is its usage site, in contrast to mammalian species. However, the actual presence of the enzymes in piscine muscle on protein level, as well as creatine synthesis in piscine muscle on metabolic level remains to be proven. We hypothesize that different amino acid metabolism in fish and mammals entails altered details of creatine metabolism during evolution.

## Materials and Methods

### Animals

The aquaculture facility of the State Research Centre for Agriculture & Fishery in Mecklenburg-Western Pomerania, Germany provided maraena whitefish (*Coregonus maraena)*, pikeperch (*Sander lucioperca*), and European perch (*Perca fluviatilis*). An experienced angler caught the Atlantic herrings from the harbor region of the Warnow River in Rostock, Germany. The Leibniz-Institute for Farm Animal Biology in Dummerstorf, Germany bred pigs (*Sus scrofa*), cattle (*Bos taurus*), and mice (*Mus musculus*). All animals represent the adult stage.

### Sampling

Except for the Atlantic herring, sampling from all other animals was implemented in the regular slaughtering process of the animal providers. The handling and sampling procedures for animals were conducted in compliance with the terms of the German Animal Welfare Act (§ 4(3) TierSchG) and approved by the internal ethics commissions of the Institute of Fisheries, State Research Centre for Agriculture and Fisheries Mecklenburg-Western Pomerania (LFA MV) and the Leibniz Institute for Farm Animal Biology. Kidney, liver, and muscle tissues were each collected from four animals of the above-mentioned species. White skeletal muscle of whitefish, pikeperch, perch, and herring were dissected 2–3 cm below the dorsal fin; *Musculus quadriceps femoris* was sampled from mice; and *Musculus longissimus dorsi* was sampled from cattle and pigs. To obtain comparable parts of the kidneys, we dissected the trunk kidney from fish species and the cortex from mammalian species. Tissue cuts were either flash-frozen in liquid nitrogen or submerged in RNAlater solution (pigs and herring). All samples were stored at −80 °C until RNA isolation.

### RNA isolation and cDNA synthesis

Tissues were homogenized in 1 ml TriZol and the RNeasy Mini Kit (Qiagen) subsequently enabled tissue-specific RNA extraction. On-column DNase treatment removed possible DNA contamination. Agarose gel electrophoresis validated RNA integrity and the NanoDrop ND-1000 determined RNA quantity. A minimum of 0.75 µg and a maximum of 1.5 µg total RNA were reverse-transcribed with Superscript II (Invitrogen) and Oligo-d(T)_24_ primers. The cDNA was purified (High Pure PCR Product Purification Kit; Roche) and diluted in 60–100 µl water, depending on RNA concentration.

### Identification of novel sequences of genes of the creatine system

We applied a PCR approach including HotStarTaq Plus DNA polymerase (Qiagen) and degenerated oligonucleotide primers. The PrimaClade^31^ tool designed primer pairs for PCR based on *GATM*, *GAMT*, and *CKM* sequences of other teleostean species available in GenBank (Supplementary Table S2). Subsequently, PCR products were cloned into TA cloning vectors (InsTAclone PCR Cloning Kit; Thermo Scientific) and the clone inserts were sequenced (Applied Biosystems 3130 Genetic Analyzer; Life Technologies). PCR amplified selected gene sequences from three individuals per species and after cloning the PCR fragments, the DNA of two clones per gene and species was sequenced, which proved fragment specificity of novel genes.

### Quantification of expression of genes of the creatine system

For the quantification of the transcript levels of *GATM*, *GAMT* and *CKM*, gene- and species-specific primers were designed (Supplementary Table S3). We analyzed the samples with the SensiFast SYBR No-ROX Kit (Bioline) on the LightCycler96 system (Roche) according to the following program (40 cycles): initial activation, 95 °C, 5 min; denaturation, 95 °C, 15 s; annealing 60 °C 10 s; elongation 72 °C, 20 s; and quantification 75 °C, 5 s. Afterwards we performed a melting curve analysis which proved the PCR products’ integrity in addition to visualizing the samples in 2% agarose gels. Copy numbers of the target genes were titrated against a dilution series (10^6^–10^3^ copies) of specific PCR amplicons serving as external standards and were then normalized against the geometric mean of the respective reference genes. The following reference genes were selected: *EIF3K* and *MTG1* for cattle^32^; *TOP2B*, *HSPCB* and *YWHAZ* for pigs^33^; *EEF2* and *RPL38* for mice^34^; *EEF1A1b*, *RPL9* and *RPL32* for maraena whitefish^35^; *ACTB* and *EEF1A* for perch^36^; *RPL38*^37^ and *EEF1A* for pikeperch and *EEF1A*^38^ and *RPL8* for herring. In addition, the copy number was normalized to an RNA input of 100 ng. An ANOVA followed by Holm-Sidak-Post-Hoc tests (Systat Sigmaplot 11) evaluated statistical significance between the data sets. The heatmap.2-function of the R gplots-package (z-transformation) generated heatmaps of logarithmized relative gene expression values. A calculation of clusters based on the UPGMA-method (centered Pearson-correlation-method) were provided by the amap-package.

### RNA-Seq analysis

We searched in the Short Reads archive (SRA) of the NCBI for publicly available data of RNA-Seq projects consisting of samples from kidney, liver and muscle tissues. Available SRA files (Supplementary Table S4) were converted to fastq files using NCBI’s SRA-Toolkit. For each species the coding sequences of *GATM*, *GAMT* and *CKM* were obtained and used as reference sequences (Supplementary Table S4) when we mapped the downloaded short reads using Bowtie 2^39^. Resultant SAM-files were converted to BAM-files, sorted and indexed by SAMtools^40^. The number of mapped and unmapped hits per gene per species was obtained using the BamIndexStats function of Picard (http://broadinstitute.github.io/picard).

## Supplementary information

Supplement tables & figures
