# Supplementary material for: Evolutionary expression differences of creatine synthesis-related genes: Implications for skeletal muscle metabolism in fish
Source: Sci Rep. 2019 Apr 1;9:5429. doi: 10.1038/s41598-019-41907-6 (PMC6443941; doi:10.1038/s41598-019-41907-6)
Supplement: Supplementary file 1 — Supplement tables & figures [file 41598_2019_41907_MOESM1_ESM.pdf]

Evolutionary expression differences of creatine synthesis-related genes: Implications for skeletal muscle metabolism in fish

Andreas Borchel[a, b], Marieke Verleih[a], Carsten Kühn[c], Alexander Rebl[a], Tom Goldammer[a, *]

[a] Fish Genetics Unit, Institute of Genome Biology, Leibniz Institute for Farm Animal Biology (FBN), Wilhelm-Stahl-Allee 2, 18196 Dummerstorf, Germany

[b] SLRC-Sea Lice Research Centre, Department of Biology, University of Bergen, Mailbox 7803, 5020 Bergen, Norway

[c] Institute of Fisheries, State Research Centre for Agriculture and Fisheries Mecklenburg-Western Pomerania (LFA MV), Fischerweg 408, 18069 Rostock, Germany

[*] Correspondence:

Dr. Tom Goldammer, Fish Genetics Unit, Institute of Genome Biology, Leibniz Institute for Farm Animal Biology (FBN), Wilhelm-Stahl-Allee 2, 18196 Dummerstorf, Germany;

Telephone +49 38208 68 708; Fax: +49 38208 68 702; E-mail: tom.goldammer@uni-rostock.de

ORCID iD: http://orcid.org/0000-0003-1215-5504

Table S1. Gene expression of *SLC6A8* in tissues of different species, determined by RNA-Seq.

Species	Reference gene	Length (bp)	CPM kidney	CPM liver	CPM muscle
Rat	NM_017348.2	3910	400.8	39.9	161.4
Sheep	ERR489264	3164	19.2	1.6	6.4
Human	NM_005629.3	3580	57.5	0.4	53.5
Rainbow trout	XM_021566996.1/	6458/	12.3/	5.9/	340.8/
	XM_021566943.1	2668	4.6	2.0	0.4
Zebra mbuna	XM_004568184.3: 1277-3295	2019	37.4	2.8	17.0
Pike	XM_013132722.2	3431	0.9	0.1	4.0

Table S2. Sequences used for the deduction of degenerated primers.

Fish species	Gene		
	GATM	*GAMT*	*CKM*
Anoplopoma fimbria	GAJJ01047113	BT082179	
Chaenocephalus aceratus			AY161316, AY161314
Haplochromis burtoni	XM_005943275	XM_005934883	
Maylandia zebra	XM_004543456	XM_004564826	
Neolamprologus brichardi		XM_006792039	
Oncorhynchus mykiss	HG315738	HG315739	HG315740
Oreochromis mossambicus			AY034097
Oreochromis niloticus		XM_003449022	
Oryzias latipes	XM_004066917	XM_004067912	
Pundamilia nyererei	XM_005753030	XM_005730241	
Takifugu rubripes	XM_003969870	XM_003974307	
Xiphophorus maculatus		XM_005800850	

Table S3. Primers used in this study.

Species	Gene	Forward Primer 5'-3'	Reverse Primer 5'-3'
Pisces[1]	*GATM*	GGMATGTATGCKGCCATGC	CGCATCCAYTCAATYCCC
	GAMT	AGGAGCACTGGATCATYGAR	TTRTTGAAGGAATAGTATTTGCATTC
	CKM	TGRTCTGGGTGAAYGAGGA	CTSTCCCTTCTCCAKCTTCTTCT
Corgenous maraena	*GATM*	GCTGTACGATCAGGAGTACCC	CATCCACTCAATCCCCATGTAG
	GAMTa	GTGGTGTCCTCACCTACTGC	TTTGCATTCGCTTGGGGGAAC
	GAMTb	CTGAAGCCCAGTGGCGTTCT	GGTTGTGGTGCTAATCATCTCC
	CKM	CGTCTGCAGAAGCGTGGCAC	TGCTGTCGATGGCCTCTCCC
	EEF1A1	CCTCCACTTGGTCGTTTCG	CGCAGGATGTAGGGCAGCAGA
	RPL9	ACCACATCAACCTGGAACTCA	CGCATCTTGTAACGGAAACC
	RPL32	CAGGCGGTTTAAGGGTCAG	ATCTCAGCAGCATGGGTC
Clupea harengus	*GATM*	ACCATGTCTGATGAGCTGTATGA	TGTAACCTGACTCCTCTGTACAA
	GAMT	CAACCACTTTGACGGCATTCTG	ATTTACCCTTCAGCAGCTCTCC
	CKM	GCAAAAGATTGAGGCCATCTTCA	TGCAGACGCAGCCTTTTCAGG
	EEF1A1	GGCTTCAACATTAAGAACGTGTC	GGTGCATAGCCAGCACTGATC
	RPL8	TAAGGGAATCGTGAAGGACATCA	TGGGCCTTCTTGCCACAGTAG
Perca fluviatilis	*GATM*	AAACGGTGCCAAATGGACCACT	CTCGAATGAAGTCAGCAGCATC
	GAMT	CAGACTTCAACATCCTGTGAGC	TCGTCCCTCTGAAGGGCCTTT
	CKM	GCGTTGGCCTGAAGAAGATTGA	TCTCCTCGAACTTGGCGTGTG
	EEF1A1	GGAAATTCGTCGTGGATACG	GGGTGGTTCAGGATGATGAC
	ACTB	ACCTTCTACAACGAGCTGAGAGTT	AGTGGTACGACCAGAGGCATAC
Sander lucioperca	*GATM*	CACTATGGCCGATGAGCTGTAT	ATTTGTAACCTGACTCCTCTGGA
	GAMT	TCGCCCCAGGAGGTCAGGTT	TCCCACACTTCCAGATAACCAC
	CKM	CCCACGGAGGCGGTGTCCA	ACGAGCATCTGGGCTACATCC
	RPL8	GTTATCGCCTCTGCCAC	ACCGAAGGGATGCTCAAC
	EF1A	ACATGCTGGAGACCAGTGAGAA	TCCAATACCGCCGATTTTGTAGA
Mus musculus	*GATM*	TACTCCTCCAACACCAGTCATC	CTGATACCCAGCTTCTCAAACAT
	GAMT	TTCGCGCCCGGCGAGGACT	AGACCCGGCCCCCTCTGGA
	CKM	ACGTGAAGCTGGCGAACCTGA	CTGTTCGACTTCGGATGAGCC
	RPL38	CCGGCGGAAGGATGCCAAGT	ACTGCCAAACCCGGGGGTAG
	EEF2	CCGGAACATGTCAGTCATCGC	GATGGCGGTGGATTTGATTGTG
Bos taurus	*GATM*	ACTCCTCCAACACCAATCATCC	ACTGATACCCAGCTTTTCAAACAT
	GAMT	CACTTTGATGGGATCCTGTACG	TGGTGGTGATGTCTGAATACTTG
	CKM	CCGTGGCATCTGGCACAATGA	CGGGTGGCCGGCTTTCTTGA
	EIF3K	CCAGGCCCACCAAGAAGAAC	GACATGGCAGATAAATTTTCGG
	MTG1	CTGGCTGACTTCCTTCTCTAC	CCGTCAGCACCTTCACCTTC
Sus scrofa	*GATM*	CCTGATTTTGAGTCTACGGGTTT	CCCACGGCGGAAGTAGTCTTT
	GAMT	TGCAATGACGGCGTCTTCCAG	CCTCAGACAGCGGGTACGTG
	CKM	AGAACCTCAAGGGTGGAGACG	CTTGAACTCTCCCGTCAGGCT
	TOP2B	ATGATGCTGGTGGCAAACACTC	TTATGAGAAGCCTCCCGCACAT
	HSPCB	GCACTCACAGTTCATAGGTTATC	ATCTGAGCCCACATCTTCAATCT
	YWHAZ	GTACTGTCTCTTTTGGAAAAGTTC	TGATTTCAAAAGCTTCCTGGTATG

[1]degenerated primers for various fish species

Table S4. Sources for RNA-Seq data and reference sequences.

Species	RNA-Seq data			Reference sequence		
	kidney	liver	muscle	*GATM*	*GAMT*	*CKM*
Anolis carolinensis	SRR579557	SRR391651	SRR579559	XM_003230613	XM_003230422	XM_008125032
Balaena mysticetus	SRR1685390	SRR1685415	SRR1685388	XM_007169721	XM_007176625	XM_007168131
Balaenoptera acutorostrata	SRR919295	SRR919296	SRR922171	XM_007169721	XM_007176625	XM_007168131
Bos taurus	ERR514720	ERR514717	ERR514718	NM_001045878	NM_001038544	NM_174773
Callithrix jacchus	SRR1758983	SRR1758984	SRR1758987	XM_002753420	XM_002761531	XM_008988218
Callorhinchus milii	SRR513760	SRR514105	SRR514104	XM_007905550	XM_007898733	XM_007885227
Esox lucius	SRR1228724	SRR1228725	SRR1228726	XM_010895950	NM_001304086	XM_010873709
Ficedula albicollis	ERR168754	ERR168726	ERR168764	XM_005052064	XM_005060155	XM_005062927
Gallus gallus	SRR594503	SRR594504	SRR594506	NM_204745	XM_001234062	NM_205507
Homo sapiens	SRR1957192	SRR1957193	SRR1957201	NM_001482	NM_000156	NM_001824
Latimeria chalumnae	DRR002308	SRR387384	DRR002313	XM_005989627	XM_005999865	XM_005991595
Maylandia zebra	SRR385837	SRR385834	SRR385833	XM_004543456	XM_004564826	XM_004569613
Mus musculus	SRR1158575	SRR1158583	SRR1158599	NM_025961	NM_010255	NM_007710
Oncorhynchus mykiss	ERR324378	ERR324372	ERR324368	HG315738	HG315739	HG315740
Oreochromis niloticus	SRR391680	SRR391688	SRR391702	XM_003458201	XM_003449022	XM_003456381
Ovis aries	ERR489260	ERR489264	ERR489244	XM_004010649	XM_012116137	XM_012190548
Pan troglodytes	SRR1758924	SRR1758924	SRR1758930	XM_001163621	XM_009434279	XM_001163660
Rattus norvegicus	SRR594431	SRR594432	SRR594434	NM_031031	NM_012793	NM_012530
Saimiri boliviensis	SRR500947	SRR500946	SRR500943	XM_003928903	XM_010332444	XM_003940241
Sus scrofa	ERR209000	ERR208927	ERR208926	NM_001128442	XM_003353975	NM_001129949
Xenopus laevis	SRR1187000	SRR514105	SRR1187003	NM_001086230	NM_001087223	NM_001086604

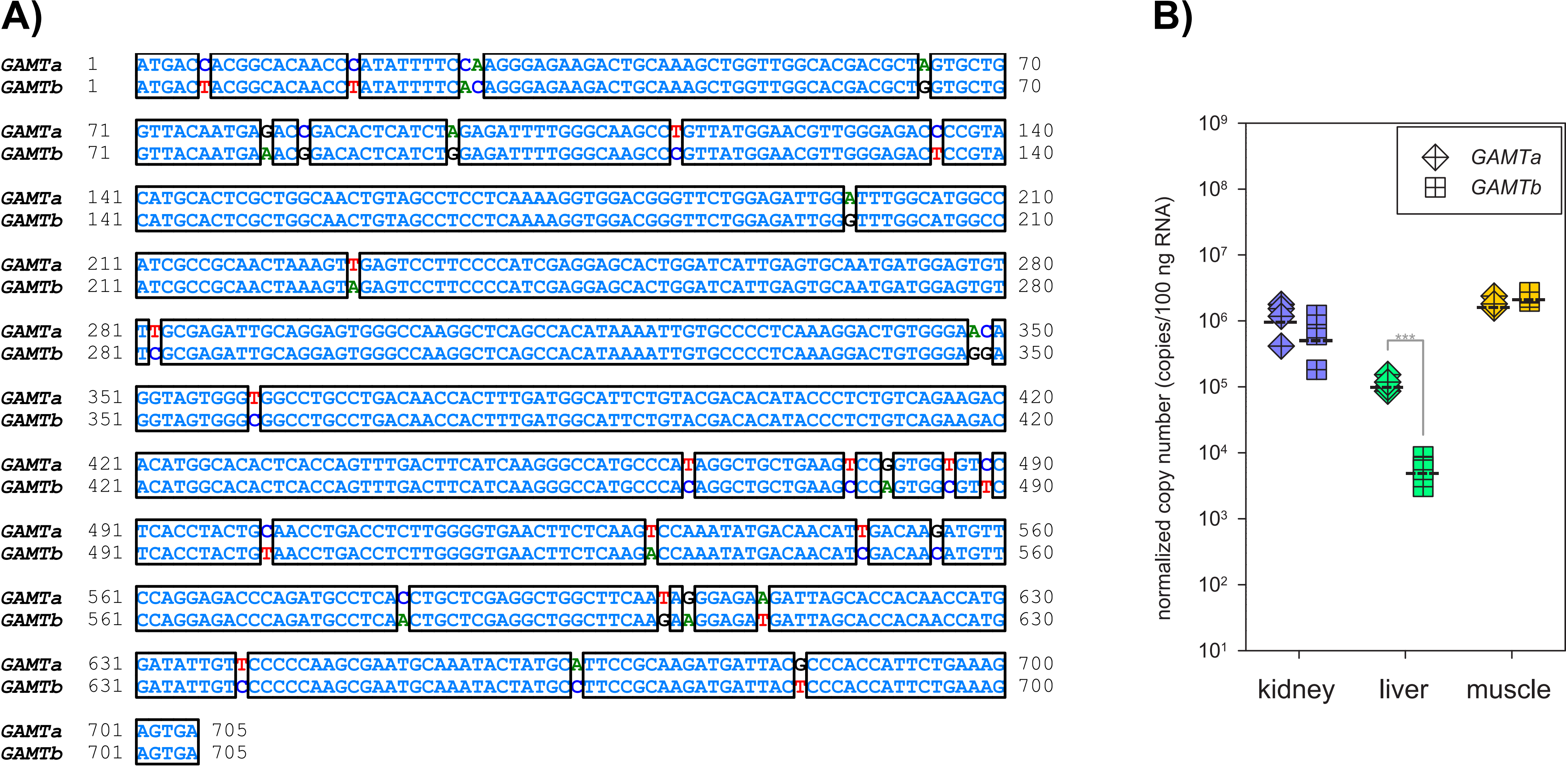

Figure S1. *GAMTa* and *GAMTb* of maraena whitefish. A) Sequence alignment of the coding regions of both *GAMT* variants. Identical bases are shadowed blue. B) Expression levels of both variants in kidney, liver and muscle. Values for *GAMTa* are the base for the whitefish *GAMT*-values in Fig. 3C. Asterisks mark significant differences between groups (*p≤0.05, **p≤0.01, ***p≤0.001).

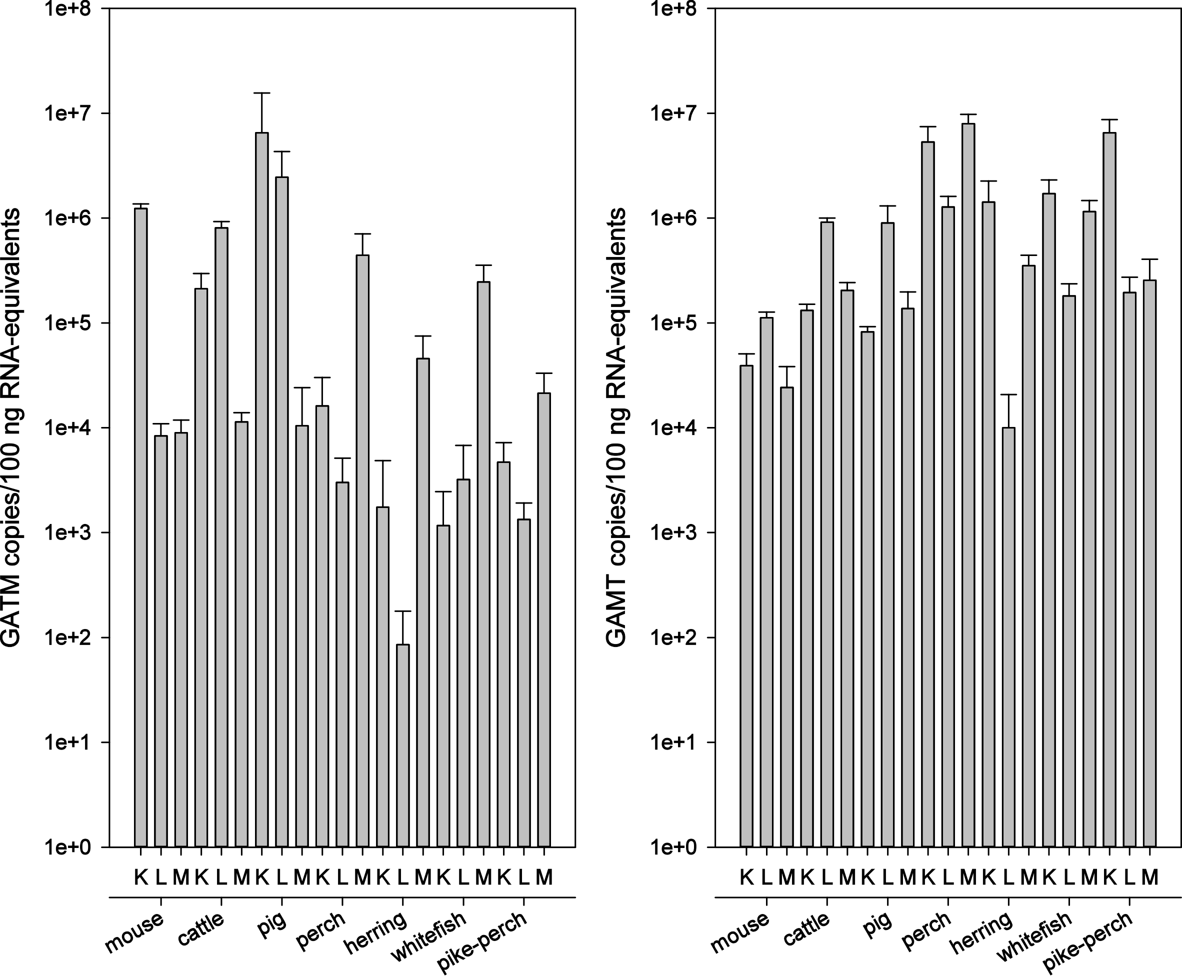

Figure S2. Absolute copy number of *GATM* and *GAMT*.

Copies of *GATM* or *GAMT* per 100 ng RNA-equivalent in the reaction. The copy numbers were directly calculated from ct values and the respective absolute standard curves and were normalized to the input of RNA into the cDNA reaction.

No normalization to reference genes was applied here.

K=kidney, L=liver, M=muscle. Values are means + standard deviation
